# The cytological changes of tobacco zygote and proembryo cells induced by beta-glucosyl Yariv reagent suggest the involvement of arabinogalactan proteins in cell division and cell plate formation

**DOI:** 10.1186/1471-2229-12-126

**Published:** 2012-08-01

**Authors:** Miao Yu, Jie Zhao

**Affiliations:** 1 State Key Laboratory of Hybrid Rice, College of Life Sciences, Wuhan University, Wuhan, 430072, China

**Keywords:** Arabinogalactan protein, *Nicotiana tabacum* L., β-GlcY reagent, Zygote, Proembryo, Cell wall

## Abstract

**Background:**

In dicotyledonous plant, the first asymmetric zygotic division and subsequent several cell divisions are crucial for proembryo pattern formation and later embryo development. Arabinogalactan proteins (AGPs) are a family of extensively glycosylated cell surface proteins that are thought to have important roles in various aspects of plant growth and development, including embryogenesis. Previous results from our laboratory show that AGPs are concerned with tobacco egg cell fertilization and zygotic division. However, how AGPs interact with other factors involved in zygotic division and proembryo development remains unknown.

**Results:**

In this study, we used the tobacco in vitro zygote culture system and series of meticulous cell biology techniques to investigate the roles of AGPs in zygote and proembryo cell division. For the first time, we examined tobacco proembryo division patterns detailed to every cell division. The bright-field images and statistical results both revealed that with the addition of an exogenous AGPs inhibitor, beta-glucosyl Yariv (beta-GlcY) reagent, the frequency of aberrant division increased remarkably in cultured tobacco zygotes and proembryos, and the cell plate specific locations of AGPs were greatly reduced after beta-GlcY treatment. In addition, the accumulations of new cell wall materials were also significantly affected by treating with beta-GlcY. Detection of cellulose components by Calcofluor white stain showed that strong fluorescence was located in the newly formed wall of daughter cells after the zygotic division of *in vivo* samples and the control samples from in vitro culture without beta-GlcY treatment; while there was only weak fluorescence in the newly formed cell walls with beta-GlcY treatment. Immunocytochemistry examination with JIM5 and JIM7 respectively against the low- and high-esterified pectins displayed that these two pectins located in opposite positions of zygotes and proembryos *in vivo* and the polarity was not affected by beta-GlcY. Furthermore, FM4-64 staining revealed that endosomes were distributed in the cell plates of proembryos, and the localization pattern was also affected by beta-GlcY treatment. These results were further confirmed by subsequent observation with transmission electron microscopy. Moreover, the changes to proembryo cell-organelles induced by beta-GlcY reagent were also observed using fluorescent dye staining technique.

**Conclusions:**

These results imply that AGPs may not only relate to cell plate position decision, but also to the location of new cell wall components. Correlated with other factors, AGPs further influence the zygotic division and proembryo pattern establishment in tobacco.

## Background

Embryogenesis is a fundamental developmental event in the life cycle of flowering plants. In higher plants, embryogenesis consists of two major phases: morphogenesis and maturation. Morphogenesis involves the establishment of the embryo’s body plan, while maturation includes cell expansion and accumulation of storage macromolecules prepared for embryo desiccation and germination as well as early seedling growth [1-3]. Embryogenesis originates from the zygotic asymmetric division which results in the formation of a small cytoplasmically-dense apical cell and a larger vacuolated basal cell [4,5]. These two distinct-sized daughter cells have different cell fates: the apical cell differentiates into an embryo proper that develops into most of the mature embryo, while the basal cell divides into the hypophysis and the suspensor [1]. The hypophysis contributes to the formation of the root meristem within the embryo proper, while the suspensor is a highly specialized, terminally differentiated embryonic organ that plays structural and physiological roles in embryo development, and degenerates at the end of embryogenesis [6-8]. The cause of the different developmental pathways of apical and basal cells remains to be researched.

The crucial concerns in plant embryogenesis research are unraveling the mechanisms that operate the processes of embryonic body plan establishment and different organ specification. The experimental manipulation for embryogenesis of angiosperms is difficult, particularly at the early stage when the embryo develops deeply inside maternal tissues [9]. In recent years, the inaccessibility of some plant embryos has been overcome. Combined with the in vitro culture system, the isolated zygotes simulate normal developmental patterns and permit direct molecular analysis at any of the early embryonic stages [10-14]. In the past few years, in our laboratory, the fertilized ovules [15,16], zygotic embryos [17,18] and even isolated zygotes [15,19] were in vitro cultured and used to study developmental events of different staged embryos. Compared with *Arabidopsis thaliana*, zygotes and proembryos in tobacco provide an ideal model system for investigating early embryo development, since they are relatively larger than those of *Arabidopsis thaliana* and can be easily isolated [15]. Recently, we extracted mRNAs from tobacco apical and basal cells to generate cDNA libraries and investigated the transcript profiles of the two daughter cells from zygotes by an expressed sequence tag analysis [20]. The strategy of combining an in vitro culture system with genetic and molecular techniques should allow us to obtain new insight into early embryo development events and embryo cell fate decision in flowering plants.

Angiosperm embryo development follows a predictable pattern and numbers of cell divisions [3,21]. In higher plants, cytokinesis is the process of separating cytoplasm through the formation of a new plasma membrane and cell wall between daughter cells [22,23]. The major stages of plant cell division contain preprophase band (PPB) formation, phragmoplast (PHP) emergence, cell plate expansion and new cell wall location. Recent studies revealed that endocytic delivery of the cell surface material significantly contributes to cell plate formation during plant cell division [22]. New cell wall components, such as pectin and wall-associated proteins (e.g. arabinogalactan proteins-AGPs), are transferred to the margin of the cell plate via endosomes [24-26]. Eventually, the expanding cell plate fuses with the parental plasma membrane at the division site, and then separates the two daughter cells.

AGPs are a large family of highly glycosylated proteins thought to be involved in plant growth and development [27-32]. In *Arabidopsis*, the organ-, tissue- and cell-type specific expression patterns indicate that AGPs are markers of cell identity and fate decision [33-35]. β-glucosyl Yariv (β-GlcY) reagent is used as an AGP inhibitor to determine AGP function by specific binding and blocking [36,37]. Although the precise effect of the β-GlcY is unknown, it is possible that the formation of large complexes at the cell surface prevents the normal assembly of molecules into cell wall [38]. In our laboratory, the previous studies have revealed that β-GlcY treatment can change the first zygotic division pattern from asymmetric to symmetric [15]. The in vitro tobacco zygote culture system with β-GlcY treatment provided us with an ideal model which might shed light on our research in the early proembryo development of higher plants. In order to delve further into this important developmental event, the molecular and cellular studies were both carried out. Recently, our transcriptome study on β-GlcY treated zygotes and two-celled proembryos also revealed that β-GlcY affected the expression of some genes related to zygotic division and proembryonic development [39].

As a continuous study of the previous work, in this paper, we used the tobacco in vitro zygote culture system and series of meticulous cell biology techniques to investigate the roles of AGPs in zygote and proembryo cell division. We adopted β-GlcY reagent to perturb AGPs function for in vitro cultured zygotes and proembryos of tobacco (*Nicotiana tabacum* L.). Both immunofluorescence detection with antibody JIM13 and staining with the β-GlcY reagent showed that AGPs distributed in the new cell plate during normal in vitro zygotic division. Cellular staining with Calcofluor white (CW) reagent (for observing cellulosic components), and fluorescent labeling with JIM5 and JIM7 monoclonal antibodies (for detecting low-esterified and high-esterified pectins, respectively) against both *in vivo* and in vitro proembryos demonstrated that β-GlcY treatment affected the location of cellulose deposits but not pectin polar distribution in the new cell wall. Furthermore, FM4-64 staining indicated that endocytic vesicles distributed in the cell plate when proembryo cells were dividing. The results imply that AGPs may be involved in the formation of new embryo cell plate. A possible model involving the relationship between AGPs and the other regulation factors was proposed and discussed.

## Methods

### Plant materials

Tobacco (*Nicotiana tabacum* L.) cultivar SR-1 was used as material. The plantlets grew in a glasshouse at 26 ± 1°C in a 16/8 h light/dark regime. The flowers were artificially pollinated during anthesis.

### Isolation of zygotes and proembryos *in vivo*

Zygotes and proembryos were isolated according to Li *et al.*[40]. Fertilized ovules at different developmental stages (3.5, 4, 4.5 and 5 days after pollination; DAP) were collected for isolating zygotes and proembryos. Firstly, ovules were placed into the enzymatic solution containing 10–13% mannitol, 3 mM MES, 1% cellulase R-10, and 0.8% macerozyme R-10, pH 5.7 for 30 min at 25°C. Then, ovules were gently ground with a small glass pestle, and embryo sacs were dissociated from the macerated ovules. After a short-term treatment of an enzymatic solution containing 13% mannitol, 3 mM MES, 0.25% cellulose R-10 and 0.2% macerozyme R-10, pH 5.7, zygotes or proembryos were isolated from embryo sacs by microdissection, and transferred into fixing solution for further experiments.

### Zygote culture in vitro

The detailed procedure of zygote culture in vitro was performed according to Qin and Zhao [15]. In zygote culture, β-GlcY reagent (Biosupplies Pty Ltd) was added to medium at a final concentration of 50 μM, and control tests performed by omitting the reagent. The medium was composed of the MS main salts plus KM8p minimal salts, organic elements, and vitamins (Sigma) (pH 5.8), and supplemented with 0.3 mol/L sucrose, 0.15 mol/L mannitol, 0.1 mol/L sorbitol, 1 mol/L 2,4-D, 0.5 mol/L 6-BA and 10% coconut (Sigma).

The isolated zygotes were cultured in a millicell (containing 150 μL of medium) with a semi-permeable membrane (Millicell-CM 0.4 μM PICM01250, diameter 12 mm), with the millicell placed in middle of a 35-mm Petri dish with 1.5 mL of medium containing 100–120 ovules at 4 DAP as feeder cells. After being cultured at 25°C in darkness for 0.5-3 d, the zygotes and proembryos were respectively collected for the next experiments.

### Immunofluorescent labeling of AGPs and pectins

The MAbs JIM5, JIM7 and JIM13 were generously provided by Prof. Paul Konx. JIM5 was used to recognize a polygalacturonic acid (low-esterified pectin) epitope, JIM7 a methylester-containing pectin epitope [41], and JIM13 for glycosyl moieties of AGPs. The samples were fixed gradually in a series of paraformaldehyde with increasing concentrations at 0.5, 1, 2 and 4% in phosphate-buffered saline (PBS pH 7.4, 2.68 mM KCl, 1.47 mM KH_2_PO_4_, 136.9 mM NaCl, 8.1 mM Na_2_HPO_4_, and 10–13% mannitol) for 10 min in each step. After three rinses with PBS buffer (pH 7.4), the fixed samples were incubated with the primary MAbs JIM5, JIM7 or JIM13 diluted 1:10 in antibody dilution buffer containing PBS buffer with 0.8% bovine serum albumin (BSA), overnight at 4°C. Subsequently, the samples were rinsed in PBS buffer three times (5 min each) and incubated with secondary antibodies at 1:100 concentrations in antibody dilution buffer (PBS buffer with 0.8% BSA) for 2 h in darkness at room temperature. Control samples were incubated with antibody dilution buffer omitting the primary antibody.

### Staining of cell walls

Calcofluor white ST (CW; America Cyanamid Co.) was used as a cellulosic wall component marker. CW stock solution (1% in bidistilled water, pH 10–11, adjusted with NaOH) was diluted 10 times in PBS buffer (pH 7.4) as a working solution. The fixed samples were incubated with CW working solution in darkness for 10 min, washed twice with PBS buffer and observed under a microscope.

### Staining for nuclear location

After the MAbs incubation or dye staining, the samples were incubated in 5 μg/mL 4’,6-diamidino-2-phenylindole (DAPI; Molecular Probes) dissolved in PBS buffer (pH 7.4) for 10 min, and then washed twice with PBS buffer. DAPI stock solution (1 mg/mL in bidistilled water) was stored in darkness at 4°C.

### Labeling endomembrane vesicles

The samples were incubated with 5 μg/mL FM4-64 [N-(3-triethylammoniumpropyl)-4- (p-diethylam-inophenylhexatrienyl)-phridinium-2Br; Molecular Probes] for 20 min at room temperature. The stock solution was prepared at a concentration of 1 mg/mL FM4-64 in dimethyl sulfoxide (DMSO, Amrasco; stored at −20°C) and the working solution dissolved in PBS buffer (pH 7.4) with 10–13% mannitol for labeling of endomembrane vesicles [42].

### Labeling endoplasmic reticulum

The stock solution was prepared by dissolving 1 mg DiOC_6_(3) in 1 mL of ethanol, and diluted 200 times in PBS buffer (pH 7.4) for staining samples. The fixated samples were stained with 2 μg/mL 3,3’-dihexyloxacarbocyanine iodide [DiOC_6_(3); Alexis] for 5 min and observed under confocal laser scanning microscopy (CLSM).

### Labeling mitochondria

The vital dye Rhodamine123 (Sigma) was used to observe mitochondria. Rhodamine123 was dissolved in DMSO at 1 mg/mL for stock solution. The living samples were stained by 10 μg/mL Rhodamine123 dissolved in PBS buffer (containing 10-13% mannitol at pH 7.4) for 10 min at room temperature, and washed three times with PBS buffer.

### Light microscopy and image collection

The samples were observed under an inverted microscope (Leica DMIRB), and the images recorded by a cooled CCD (Micro MAX Princeton Instruments Inc.) or a confocal laser scanning microscope (FluoView^TM^ FV1000, Olympus).

### Transmission electron microscopy (TEM)

*In vivo* samples: the isolated ovules at 3–4 DAP were fixed in 3% glutaraldehyde and PBS (pH 7.4) under vacuum at room temperature for 2 h, and then kept in fresh fixative for 4–6 h. After several rinses with PBS buffer, the samples were post-fixed in 1% osmium tetroxide at 4°C overnight, then dehydrated in a series of ethanol gradients, and embedded in Spurr resin. Resin polymerization was carried out at 65°C overnight. Ultrathin sections (60–70 nm) were cut under a Sorvall MT-6000 ultramicrotome using glass knives and collected onto Formvar-coated copper grids. The sections were examined and photographed under a transmission electron microscope (H-800; Hitachi).

In vitro samples: the zygotes and proembryos cultured in vitro were collected and fixed gradually in a series of increasing concentrations of 0.5, 1 and 2% glutaraldehyde in PBS (pH 7.4) for each step in 30 min, then embedded into 2% agar and fixed in fresh fixative for 3 h. The post-fixation, dehydration, embedding and the later procedures were performed according to the above described method.

## Result

### Pattern of early proembryo formation in tobacco

**Figure 1:**
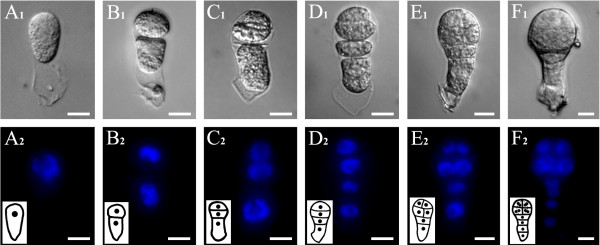
**Cell division pattern of zygote and proembryo in tobacco.** (A_1_-H_1_) Bright-field images. (A_2_-H_2_) Fluorescence images of DAPI-stained nuclei corresponding to bright-field images. (**A**_**1**_ and **A**_**2**_) A isolated live zygote at 84 h after pollination. (**B**_**1**_ and **B**_**2**_) A two-celled proembryo with a small apical cell and a large basal cell from a zygote asymmetrical division. (**C**_**1**_ and **C**_**2**_) A three-celled proembryo with a large basal cell and two-celled embryo-proper after apical cell division. (**D**_**1**_ and **D**_**2**_) A four-celled proembryo with a two-celled embryo-proper and a two-celled suspensor. (**E**_**1**_ and **E**_**2**_) A proembryo with an eight-celled embryo-proper and a two-celled suspensor. (**F**_**1**_ and **F**_**2**_) A multicellular proembryo with a four-celled suspensor. The inserted pictures in **A**_**2**_**-F**_**2**_ are schematic diagrams of zygotes and proembryos. Bars = 10 μm.

Initially, we examined early proembryo division patterns *in vivo* for tobacco. After the egg cell was fertilized, the zygote formed, subsequently elongated (Figure 1A_1_) and underwent an asymmetrical transverse division to produce a small, spherical apical cell and a larger, elongated basal cell (Figure 1B_1_). Both the apical and basal cells underwent a transverse division to generate three- and four-celled proembryos (Figure 1C_1_, D_1_). In the four-celled stage, two longitudinal divisions of the two upper cells produced an octant embryo-proper (Figure 1E_1_, F_1_). Then, the eight cells of the embryo-proper periclinally divided and gave rise to 16-celled embryo-proper (data not shown). The basal cell underwent two transverse divisions to form the suspensor, composed of a single line of four cells (Figure 1F_1_). Using DAPI staining as a guide, the nuclei in proembryos were observed, and served as a marker for rapidly determining the cell number and relative position (Figure 1A_2_-F_2_).

### Proembryo division pattern change induced by treatment of β-GlcY reagent

**Figure 2:**
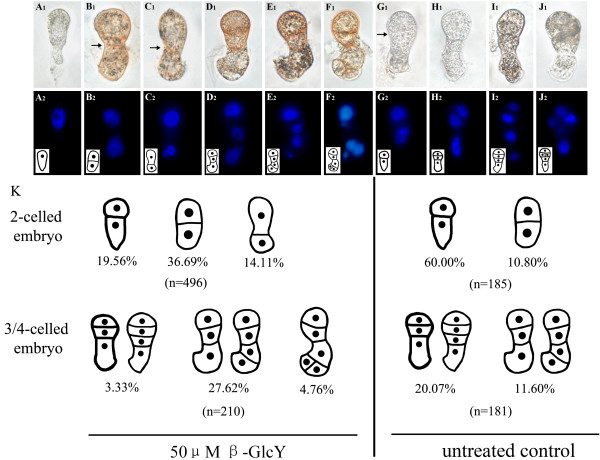
**Division patterns of tobacco in vitro cultured proembryos.A**_**1**_**-J**_**1**_ show bright-field images of an isolated zygote (**A**_**1**_), 50 μM β-GlcY treated proembryos (**B**_**1**_**-F**_**1**_) and untreated proembryos (**G**_**1**_**-J**_**1**_). The walls between apical and basal cells are indicated by arrows. (**A**_**2**_**-J**_**2**_) Fluorescence images of DAPI-stained nuclei corresponding to bright-field images. The inserted pictures in **A**_**2**_**-J**_**2**_ are schematic diagrams of zygotes and proembryos. (K) Schematics of the treated and untreated proembryos. The frequencies and numbers of the examined proembryos are noted below the drawings. Bars = 10 μm.

Tobacco zygotes were isolated and cultured in vitro to observe the changes in proembryo cell division after treatment with β-GlcY reagent (Figure 2A_1_, A_2_). In treated samples, 70.36% of zygotes (n = 496) completed the first division, compared to 70.80% in untreated samples (n = 185). After 2–3 d of in vitro culture, 37.71% of treated zygotes divided into multicellular proembryos, versus 31.67% in control culture (Figure 2K). After 50 μM β-GlcY reagent was added into the medium, the position of cell plate for the first division of zygotes (Figure 2B_1_, C_1_, B_2_, C_2_) differed from that of untreated zygotes (Figure 2G_1_, G_2_). The abnormal position of the cell division plane resulted in forming two equal cells or a larger apical cell relative to the basal cell (Figure 2B_1_, C_1_, B_2_, C_2_). In contrast, most untreated elongated zygotes asymmetrically divided into two-celled proembryos (Figure 2G_1_, G_2_), similar to the natural division pattern of zygotes *in vivo* (Figure 1B_1_). Among the 185 untreated zygotes, the frequency of the first asymmetrical division was up to 60%, but symmetric division reached only 10.80% (Figure 2K). However, the treated samples had a significantly higher percentage (approximately 50%) of abnormal two-celled proembryos compared with untreated samples (Figure 2K).

The subsequent proembryo cell divisions were also affected by the β-GlcY treatment, with the cell division plane occurring in apparently random directions. After 2–3 d of culture, nearly one-third of the zygotes divided into 3-5-celled proembryos (Figure 2K). In β-GlcY treated samples, 27.62% 3/4-celled proembryos divided aberrantly (Figure 2D_1_, E_1_, D_2_, E_2_; Figure 2K), and 4.79% proembryos became a budded structure (Figure 2F_1_, F_2_; Figure 2K), while the frequency of normal proembryos decreased to 3.33% (Figure 2K). In contrast, most untreated zygote cells normally divided as per the natural division pattern *in vivo* and formed an embryo proper (Figure 2H_1_-J_1_, H_2_-J_2_), serving as an important control. The data in Figure 2K illustrates that β-GlcY does not affect the survival rate and cell division frequency, but specifically has influence on cell plate position decision of tobacco zygotes and proembryos, suggesting that AGPs may be involved in zygote division and early proembryo pattern formation.

### The influence of β-GlcY reagent on AGPs distribution

**Figure 3:**
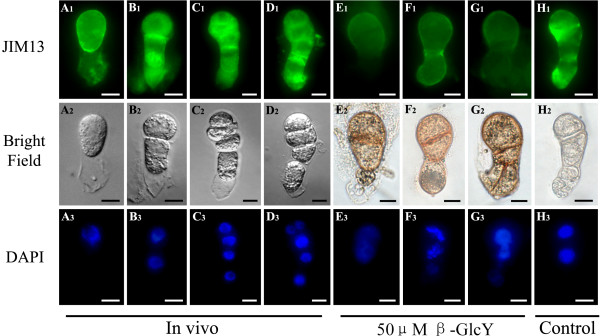
**Localization of AGPs by immunofluorescence labeling with JIM13 in zygotes and proembryos.** (**A**_**1**_**-H**_**1**_) The fluorescence images of immunolabeling with anti-AGP antibody JIM13 under a fluorescence microscope. The antibodies labeled both cell surfaces and cell plates of zygote and proembryos *in vivo* (**A**_**1**_**-D**_**1**_). In the β-GlcY treated proembryos, the immunofluorescences are remarkably weakened, especially in the cell plates (E_1_-G_1_). Under β-GlcY treatment, the cell plates of proembryos are stained into red (**E**_**2**_**-G**_**2**_). (H_1_) The immunolabeling signals in the untreated proembryos in vitro are similar to that of *in vivo* ones. (A_2_-H_2_) Bright-field images corresponding to **A**_**1**_**-H**_**1**_. (**A**_**3**_**-H**_**3**_) Fluorescence images of DAPI-stained nuclei corresponding to bright-field images. Bars = 10 μm.

By using the monoclonal antibodies JIM13 and β-GlcY reagent, we could detect the AGPs distribution in proembryos. The cell plates of proembryos were stained a red color (Figure 3E_2_-G_2_) with β-GlcY treatment, and the distribution pattern was similar to the fluorescent result of JIM13 epitopes (Figure 3B_1_-D_1_), both indicating abundant AGPs in the newly generated cell plate. Compared with *in vivo* (Figure 3A_1_-D_1_) and in vitro untreated proembryos (Figure 3H_1_), the immunofluorescence in the treated proembryo cells (Figure 3E_1_-G_1_) was remarkably weakened, especially in cell plate regions, suggesting that β-GlcY specifically bound the carbohydrate of AGPs, and then could obstruct its binding with monoclonal antibody JIM13. In addition, a negative control without primary antibody showed no fluorescence in samples (data not shown).

### The polar distribution of pectins in zygotes and proembryos *in vivo* and in vitro

**Figure 4:**
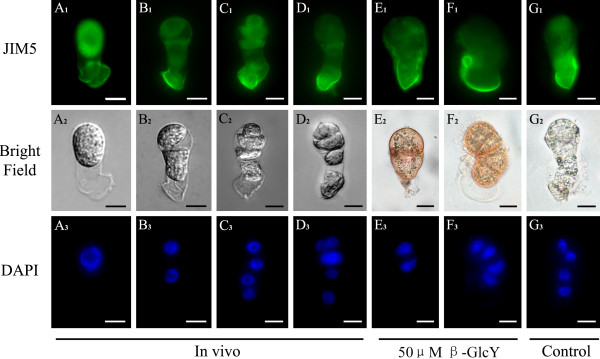
**Distribution of low-esterified pectins by immunolabeling with JIM5 in zygotes and proembryos.** (**A**_**1**_**-G**_**1**_) The fluorescence images of immunolabeling with low-esterified pectins antibody JIM5 under a fluorescence microscope. The intense green immunofluorescence localized mainly in the cell walls of basal end of zygotes and proembryos *in vivo* (**A**_**1**_**-D**_**1**_), proembryos cultured with 50 μM β-GlcY (**E**_**1**_ and **F**_**1**_) and the samples cultured in vitro without β-GlcY (G_1_). (**A**_**2**_**-G**_**2**_) Bright-field images of zygotes and proembryos corresponding to **A**_**1**_**-G**_**1**_. (**A**_**3**_**-G**_**3**_) Fluorescence images of nuclei in zygotes and proembryos by DAPI staining. Bars = 10 μm.

**Figure 5:**
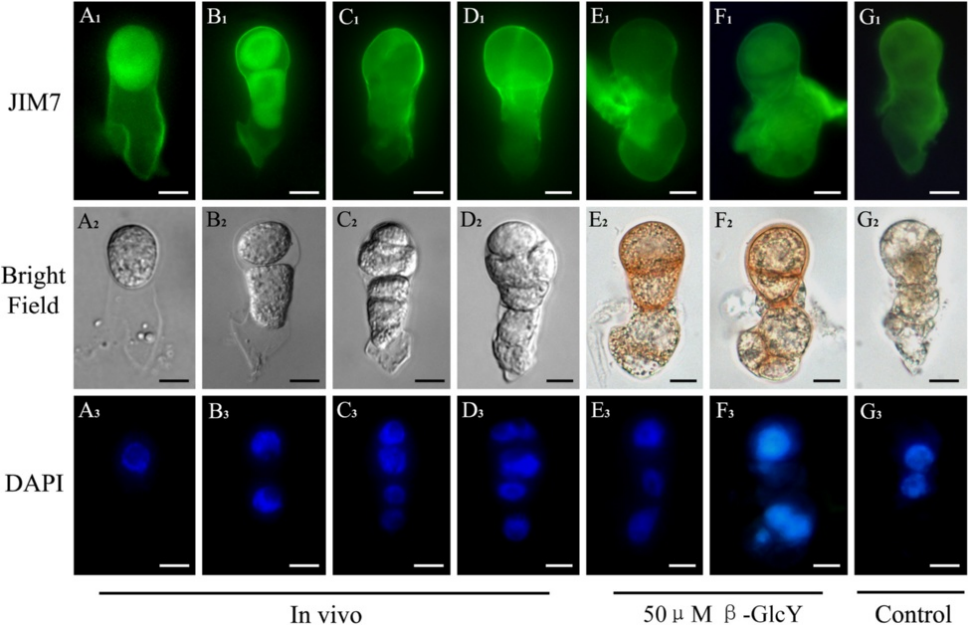
**Distribution of high-esterified pectins by immunolabeling with JIM7 in zygotes and proembryos.** (**A**_**1**_**-G**_**1**_) The fluorescence images of immunolabeling with high-esterified pectins antibody JIM7 under a fluorescence microscope. The immunofluorescence gradually localizes in the apical end of cell wall during zygotes divide into proembryos *in vivo* (**A**_**1**_**-D**_**1**_). In in vitro samples, high level immunofluorescence distributes in the whole cell walls of proembryos cultured with 50 μM β-GlcY (E_1_-F_1_) and the samples cultured without β-GlcY (**G**_**1**_). (**A**_**2**_**-G**_**2**_) Bright-field images of zygotes and proembryos corresponding to **A**_**1**_**-G**_**1**_. (**A**_**3**_**-G**_**3**_) Fluorescence images of DAPI-stained nuclei corresponding to bright-field images. Bars = 10 μm.

Monoclonal antibodies JIM5 and JIM7 bind to distinct partially methylesterified epitopes of pectic homogalacturonan (HG), with JIM5 for low-esterified and JIM7 for high-esterified pectins. In tobacco zygotes and proembryos, the two pectins displayed different distributions (Figures 4 and 5). For *in vivo* zygotes, the labeling of JIM5 showed low-esterified pectins distributed mainly in the basal end (or micropylar end) cell wall of the, but markedly weaker distribution in the cytoplasm and apical end (or chalazal end) cell wall (Figure 4A_1_). After the zygotes division, the immunofluorescence was more concentrated in the basal end of the cell walls (Figure 4B_1_-D_1_). Furthermore, in the in vitro cultured zygotes and proembryos, JIM5 immunofluorescence also showed that low-esterified pectin was mainly distributed in the basal end cell wall (Figure 4E_1_-G_1_). The distribution patterns of low-esterified pectin were similar between the treated (Figure 4E_1_, F_1_) and untreated samples (Figure 4G_1_). Thus, there was no difference in distribution of the low-esterified pectin between *in vivo* and in vitro cultured zygotes and proembryos, and we may conclude that β-GlcY did not affect the distribution of the low-esterified pectin.

In contrast with JIM5, the labeling of JIM7 displayed a different distribution pattern (Figure 5). For *in vivo* zygotes, JIM7 immunofluoresence was located in the entire cell wall and was slightly weaker in the cytoplasm (Figure 5A_1_). After zygotic division, the fluorescence of high-esterified pectin gradually concentrated to the apical end of *in vivo* proembryos (Figure 5B_1_-D_1_). In in vitro cultured proembryos (Figure 5E_1_-G_1_), the immunofluorescence showed high-esterified pectin distribution patterns were similar between the treated (Figure 5E_1_, F_1_) and untreated samples (Figure 5G_1_); however, there were differences with *in vivo* proembryos (Figure 5B_1_-D_1_). For in vitro cultured proembryos, high-esterified pectins distributed in whole cell walls of β-GlcY treated (Figure 5E_1_, F_1_) or not treated samples (Figure 5G_1_). The JIM7 immunofluorescence revealed that in vitro culture could alter the distribution pattern of high-esterified pectins.

### Cellulose accumulation change induced by treatment of β-GlcY reagent

**Figure 6:**
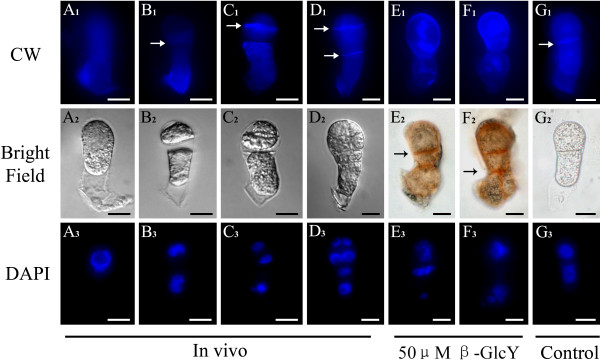
**Distribution of cellulose revealed by Calcofluor white (CW) staining in zygotes and proembryos.** (**A**_**1**_**-D**_**1**_) In the isolated zygotes and proembryos *in vivo*, the fluorescence of CW deposited both in cell surface and cell plate walls. The cell plates are highlighted by arrows. (**E**_**1**_**-F**_**1**_) With 50 μM β-GlcY treatment, the fluorescence in the newly formed cell plate walls weakens. (**G**_**1**_) The fluorescence image of untreated proembryo cultured in vitro is similar with that of *in vivo*. (**A**_**2**_**-G**_**2**_) Bright-field images of zygotes and proembryos corresponding to **A**_**1**_**-G**_**1**_. (**A**_**3**_**-G**_**3**_) Fluorescence images of DAPI-stained nuclei corresponding to bright-field images. Bars = 10 μm.

To observe the cellulose distribution in zygotes and proembryos *in vivo* and in vitro, we used Calcofluor white (CW) as a fluorescent dye. CW binds to β1-3 and β1-4 polysaccharides such as callose and cellulose, which are common components in plant cell walls. We stained both *in vivo* and in vitro samples with CW reagent and observed that the fluorescence labeling of the isolated proembryos *in vivo* (Figure 6A_1_-D_1_) and untreated proembryos in vitro (Figure 6G_1_) was deposited abundantly in the cell plate wall. In proembryos cultured with 50 μM β-GlcY reagent, however, the CW fluorescence signal of cell plates was weaker or completely absent (Figure 6E_1_, F_1_), instead, these regions were distinctly stained a red color with β-GlcY (Figure 6E_2_, F_2_). Thus the β-GlcY treatment may affect the location of newly generated cell plate wall components.

### The influence of β-GlcY reagent on endocytosis distribution

**Figure 7:**
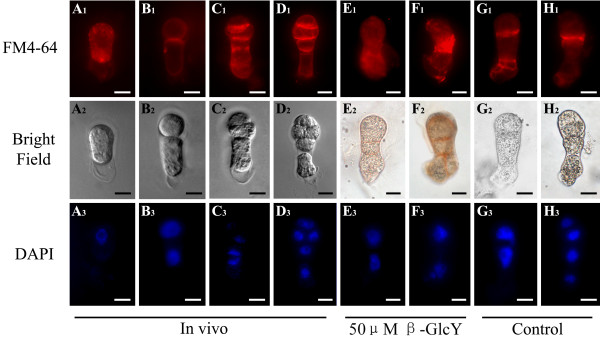
**Localization of endocytic vesicles by FM4-64 staining in tobacco zygotes and proembryos.** (**A**_**1**_**-H**_**1**_) The fluorescence images of living zygotes and proembryos stained by FM4-64. In a zygote, the fluorescence of FM4-64 evenly distributes in the whole cytoplasm (**A**_**1**_). During cell plate formation, endocytic vesicles mainly localize in the new cell plate edges (**B**_**1**_**-D**_**1**_). After treating with 50 μM β-GlcY, the fluorescence of FM4-64 evenly distributes in cytoplasm of in vitro cultured proembryos (**E**_**1**_ and **F**_**1**_). In proembryos cultured without β-GlcY, the fluorescence images are similar to that of *in vivo* proembryos (**G**_**1**_). (**A**_**2**_**-H**_**2**_) Bright-field images of zygotes and proembryos corresponding to **A**_**1**_**-G**_**1**_. (**A**_**3**_**-H**_**3**_) Fluorescence images of DAPI-stained nuclei corresponding to bright-field images. Bars = 10 μm.

A fluorescent lipophilic styryl dye, FM4-64, is often used as an authentic endocytic marker in living plant cells [43], and is taken up into the cell interior only by endocytosis and hence can gradually labels the endocytic pathway. To investigate the change in endomembrane structures, we used FM4-64 to stain the living zygotes and proembryos *in vivo* and in vitro. In the zygotes, the fluorescence of FM4-64 was equidistributed in the whole cytoplasm (Figure 7A_1_). When zygotes divided into multi-cell embryos, the signal of FM4-64 was detected extensively among the newly formed cell plates and their edges (Figure 7B_1_-D_1_). However, in β-GlcY treated proembryos, the specific signal at cell plate edges disappeared and was replaced by equidistribution or abnormal accumulation of the FM4-64 signal in the cytoplasm (Figure 7E_1_, F_1_). In addition, in the proembryos cultured without β-GlcY, the newly formed cell plates were clearly labeled by FM4-64 (Figure 7G_1_, H_1_). It’s well known that endocytic delivery of cell surface material significantly contributed to cell plate formation during cell division; hence the endosome distribution change induced by β-GlcY may suggest an important role of AGPs in cell plate formation.

### Ultrastructure characteristics in zygotes and proembryos

**Figure 8:**
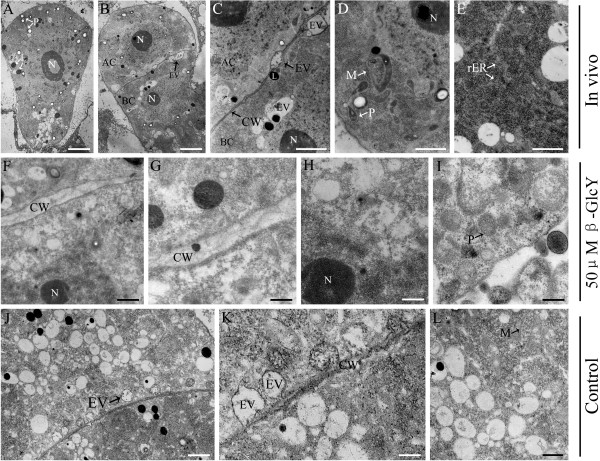
**Transmission electron micrographs of tobacco zygotes and proembryos. (A)** Ultrathin section of a zygote at 84 h after pollination. The plastids, vesicles and lipid bodies are highlighted with adjacent arrows. Bar = 3 μm. **(B)** The two-celled proembryo with an apical cell and a basal cell. Bar = 3 μm. **(C)** The local amplification of figure B shows plasma membrane and cell wall between apical and basal cells. Some endocytic vesicles localize in cell plate and are highlighted with arrows. Bar = 1 μm. **(D)** Cytoplasm in a proembryos *in vivo*. The plastids, mitochondria and rough-endoplasmic reticulum are highlighted with adjacent arrows. Bar = 1 μm. **(E)** Cytoplasm in a proembryos *in vivo*. Bar = 500 nm. (F-I) Ultrathin section images of 50 μM β-GlcY treated proembryos. After treatment with β-GlcY, the localization of endocytic vesicles in cell plates is affected (**F** and **G**). The distribution and morphology of cell organelles in β-GlcY treated proembryos are also changed (**H** and **I**). Bars = 500 nm in **(F)**; 200 nm in **(G, H and I)**. (**J-L**) Ultrathin section of in vitro cultured proembryo without β-GlcY treatment. Some endocytic vesicles localize in cell plate and the pattern is similar with *in vivo* proembryos. Bars = 500 nm in **(J)**; 200 nm in **(K and L)**. AC, apical cell; BC, basal cell; CW, cell wall; rER, rough-endoplasmic reticulum; L, lipid body; M, mitochondrion; N, nucleus; P, plastid; EV, endocytic vesicle.

The ultrastructure micrographs were acquired by transmission electron microscopy, and more details of zygotes and proembryos *in vivo* and in vitro were revealed in these pictures. In Figure 8A, the elongated, pyriform-shaped zygote *in vivo* in tobacco was highly polarized, with a large nucleus located in the chalazal end of the cell and some small vacuoles in the micropylar end, and starch-containing plastids greatly accumulated around the nucleus (Figure 8A). The first zygote division was transverse, producing a small spherical apical cell and a large basal cell (Figure 8B). In the *in vivo* two-celled embryos, there were numbers of endoplasmic reticulum (ER), plastids and mitochondria similar to the zygote (Figure 8D, E). Some endocytic vesicles were attached to the cell wall, and the others appeared nearby the cell plate region (Figure 8C), indicating that endocytic vesicles were involved in cell plate formation. In vitro untreated proembryos showed similar features to *in vivo* proembryos (Figure 8J-L). However, the β-GlcY treated proembryo cells differed with both *in vivo* and in vitro non-treated proembryos-endosomes in the cell plate were not observed (Figure 8F-I). Thus, the results suggested that AGPs distribution might be involved in endocytic vesicles location and further in cell plate formation.

**Figure 9:**
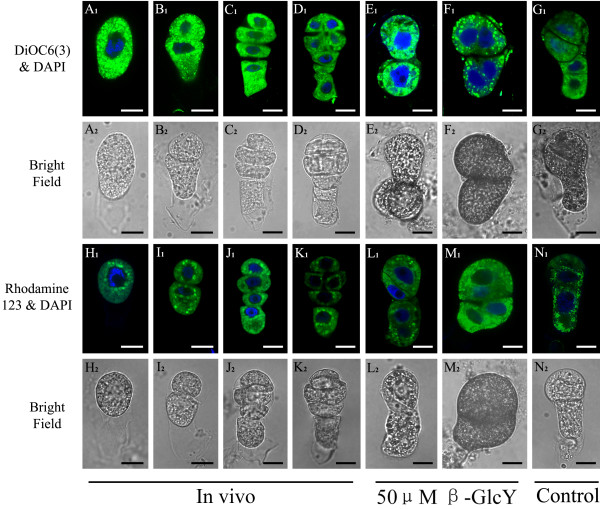
**Distribution of endoplasmic reticulum and mitochondria in tobacco zygotes and proembryo cells.** (**A**_**1**_**-G**_**1**_) The fluorescence images of endoplasmic reticulum in zygotes and proembryo stained by DiOC_6_(3) under a confocal laser scanning microscope. (**A**_**1**_**-D**_**1**_)The green fluorescence signals of DiOC_6_(3) appear equably in the isolated *in vivo* zygotes and proembryo cells. (**E**_**1**_ and **F**_**1**_) The patterm of fluorescence signals of DiOC_6_(3) were disorganized in the zygote and proembryo cells treated with 50 μM β-GlcY reagent. (**G**_**1**_) The fluorescence of DiOC_6_(3) in the untreated zygotes and proembryos cultured in vitro is similar to those *in vivo*. (**H**_**1**_**-N**_**1**_) The fluorescence images of mitochondria in zygotes and proembryo stained by Rhodamine123 under a confocal laser scanning microscope. (H_1_-K_1_) The green fluorescence of Rhodamine123 in isolated zygotes and proembryos indicates mitochondria localization around nuclei. (**L**_**1**_**-M**_**1**_) With 50 μM β-GlcY treatment, the distribution of mitochondria in proembryos is disordered. (**N**_**1**_) The fluorescence of untreated samples cultured in vitro is similar to those *in vivo*. The nuclei are stained by DAPI and display blue fluorescence in **A**_**1**_**-N**_**1**_. (**A**_**2**_**-N**_**2**_) The bright-field images of zygotes and proembryos corresponding to Figure A_1_-N_1_. Bars = 10 μm.

In β-GlcY treated proembryos, we also found that cell-organelles, such as plastids and mitochondria, showed morphological changes, and the number of ribosome decreased (Figure 8F-I). Then, we observed the ER and mitochondria distribution using the fluorescent dyes DiOC_6_(3) and Rhodamine123, respectively (Figure 9), which gave similar results with those observed under transmission electron microscopy. The ER was evenly distributed in the cytoplasm of *in vivo* zygotes and proembryos (Figure 9A_1_-D_1_). In vitro culture could change ER distribution either with (Figure 9E_1_-F_1_) or without β-GlcY treatment (Figure 9G_1_); however, the ER distribution was much more disordered in treated samples than those untreated. The mitochondria were distributed around the nucleus in cytoplasm of *in vivo* zygotes and proembryos (Figure 9H_1_-K_1_). For in vitro cultured samples, it displayed a disorderly distribution of mitochondria after β-GlcY treatment (Figure 9L_1_-M_1_), while mitochondria localization in untreated proembryos were similar to those *in vivo* (Figure 9N_1_). Above all, β-GlcY not only affected the cell plate formation but also had influence on cell-organelle distribution, suggesting roles of AGPs in these processes.

## Discussion

Angiosperm embryo development follows a predictable pattern of planes and numbers of cell divisions [3,21,42]. In the present study, we firstly examined the detailed division events of normal proembryo development in tobacco (Figure 1). Intriguingly, we also found that there are differences between *N. tabacum* and *Arabidopsis* in the initial division patterns of apical cells and in suspensor cell numbers. In tobacco, the resultant zygote elongated and then divided asymmetrically to form two daughter cells of different sizes (Figure 1A_1_, B_1_). The small apical daughter cell firstly underwent one transverse and then two rounds of longitudinal divisions to give rise to an eight-celled embryo proper (Figure 1F_1_). While in another typical dicot *Arabidopsis*, the apical cell undergoes two longitudinal and then one transverse division to form an octant embryo proper [44]. Concurrently, the basal cell divides transversely to form a suspensor of 3–4 cells in tobacco, compared to 6–7 cells in the *Arabidopsis* suspensor.

The well-established experimental system of isolation and culture of tobacco zygotes and proembryos is propitious to investigating the early events and mechanism of dicotyledonous plant embryogenesis. Recently, some transcriptome research in tobacco zygotes and proembryos provided much data in zygote gene activation (ZGA) and embryo polarity establishment [20,45,46]. We also use the in vitro culture system with β-GlcY treatment, which could provide symmetrically divided proembryos compared with the symmetric ones *in vivo*, to study the early proembryo development. Besides the cellular study results we have shown in this paper, the molecular methods were also applied in order to uncover the mechanisms how zygote initiate the first asymmetric division. Recently, our transcriptome studies on β-GlcY treated zygotes and two-celled proembryos have identified some candidate genes might related to zygotic division and proembryonic development [39]. Therefore, combination of cytological and molecular approaches with genetic methods will give more investigation outcomes in early plant embryo development and fill more research gaps.

### Possible roles of AGPs in the proembryonic cell fate decision

In dicotyledons, such as *Arabidopsis* and tobacco, the polarity of the proembryo was established as early as the first asymmetric zygote division. After that, the two daughter cells undergo distinct developmental fates. Previous researches have shown that the apical and basal cells were different in shape, size, cytoplasm and even in gene transcriptions [20,42,44]. In our studies, with β-GlcY treatment, the proper zygotic division and subsequent cell divisions of the proembryo were affected, and formed aberrant proembryos which had lost morphological polarity (Figure 2). Furthermore, the detailed effects induced by β-GlcY were observed by photomicrograph and electron microscope (Figure 2 and 8), and the results showed that the typical characteristics of apical cell, like relatively small size and dense cytoplasm were changed, concurrently, their developmental pathway deviates from the initial destiny. Some studies on zygotic embryogenesis of many different plants had also revealed that AGPs involved in maintenance of meristem cells, and the other researched in somatic embryogenesis also showed the involvement of AGPs in the induction of embryonic cells [15-18,47-50]. In microspore embryogenesis, it was also revealed that AGPs might be able to induce embryogenic proficiency [51]. In *Arabidopsis*, the result of our lab had displayed that β-GlcY treatment can change the shoot apical meristem cells in torpedo-stage embryo from typical meristem cells into differentiated cells, which contained more starch grains and larger vacuoles [18]. In early embryo stage of tobacco, we also found that β-GlcY treatment not only affected the cell division planes and relatively cell size, but also influenced subcellular structures (Figure 2 and 8), such as organelles abundance and distribution, which were the typical characteristics to distinguish the cell differentiation degree. Recently, our another research by comparative transcriptional analysis in β-GlcY treated and *in vivo* untreated two-celled proembryos indicated that the expression of some transcripts related to zygotic cell division and development was significantly different [39]. Above all, the significant influence of β-GlcY treatment on the developmental fate of zygotic and somatic embryo cells suggest that AGPs might play important roles in these processes. However, the details of how AGPs are involved in proembryo cell fate decision still need more research.

### β-GlcY reagent affects new cell wall components accumulation in tobacco proembryo

In the proembryos, like other organs of higher plants, the position of new cell plate determines whether the division is symmetrical or asymmetrical, and affects the subsequent differentiation and development of daughter cells. In our study, we found that AGPs were abundant in tobacco zygotes and proembryos, especially in the newly formed cell plate region (Figure 3). These results reveal that AGPs may be involved in cell plate location, which is pivotal for normal proembryo development of plants. Some studies have revealed that endocytic delivery significantly contributes to cell plate formation during plant cell division [22]. It’s via endosomes that new cell wall components such as pectins and cell-wall-associated proteins (e.g. AGPs) are transferred to the margin of the cell plate [24-26]. Therefore, endosomes are involved in the execution of cytokinesis and the assembly of the cell wall. Previous research in lily pollen tubes showed that the secretory endosomes in the tips of pollen tubes were destabilized by β-GlcY reagent, and normal incorporation of cell wall components was prevented, which inhibited the tube tip growth [38]. In the present study, the accumulation of cellulose in newly formed cell plates was blocked by β-GlcY reagent (Figure 6). Furthermore, staining of endocytotic delivery with FM4-64 showed that the distribution of endosomal vesicles in cultured proembryo cells was disrupted by adding β-GlcY reagent (Figure 7). Our study indicates the the correlation of endosomal vesicles and cell plate formation, and suggest the involvement of AGPs in cell wall material localization.

Plant cells wall are highly sophisticated structures consisting of diverse polysaccharides, such as cellulose, hemicellulose, pectin, and structural proteins [24-26], and pectins are the major components of the primary cell wall in dicotyledonous plants [52]. Some researchers have shown that AGPs might relate with pectin distribution in plant cells [18]. However, we were surprised to find that pectins with different degrees of esterification displayed distinct polar distribution in the cell wall of zygotes and proembryos, and the polarity was not affected by β-GlcY treatment (Figures 4 and 5). Currently, little is known about the biosynthesis mechanism of pectins. It has been assumed that pectins are synthesized and methyl-esterified in the Golgi and secreted to the cell wall in a highly methyl-esterified form, and are subsequently de-esterified by pectin methylesterases [53]. Immunolocalization in pollen tube showed that high-esterified pectins were exclusively present in the apical region of pollen tube walls, while low-esterified pectins widely distributed in the entire the tubes [54]. In addition, it was reported that the de-esterified pectins can bind Ca^2+^ ions and reinforce the cell wall structure [55]. In our study, JIM5 labeled low-esterified pectins were mainly localized in the basal end of tobacco zygote and proembryo cell wall *in vivo*; conversely, JIM7 recognized high-esterified pectins mainly distributed in the apical end of the cell wall (Figures 4A-D and 5A-D). Combined with previous studies, our observation in tobacco proembryos *in vivo* suggest that JIM7 recognized high-esterified pectins may be involved in the polar growth of proembryos, while the basal distributed low-esterified pectins may structurally rigidify the cell wall. However, the detailed mechanism of this interesting polar distribution of pectin in tobacco proembryos still need more research. Furthermore, in contrast to low-esterified pectins, the distribution patterns of high-esterified pectins differed between *in vivo* and in vitro cultured samples (Figure 5). Thus the pattern change of high-esterified pectins may be also associated with in vitro culture, not only with β-GlcY treatment.

### The possible model of AGPs involved in cell plate formation during proembryo development

In the brown algae *Fucus*, the first asymmetrical cell division is associated with targeted secretion of cell wall components. Actin microfilaments and an intact cell wall are both required for directional vesicle secretion to stabilize its polar axis [56-58]. Some researchers demonstrated that β-GlcY treatment depolymerized and disorganized cortical microtubules in Brigit Yellow-2 (BY-2) cells and affected the organization of the F-actin [59,60]. Another group reported that cross-linking of AGPs with β-GlcY would result in inhibition of tobacco cell elongation, and proposed that AGPs were probably important regulators of plant cell growth [61]. It is well known that endosomal movement in plant cells is dependent on transportation of cytoskeleton network [62]. The present study suggests that the β-GlcY reagent entered the cell wall, bound to AGPs and formed AGP-Yariv complexes, thereby disrupting interactions of AGPs with other molecular components in the cell wall and plasma membrane, and finally obstructing endocytotic delivery and cytoskeleton transportation [63,64]. The other studies showed that AGPs may be involved in cellulose deposition in plant cells [65]. The previous works of our laboratory showed that the fasciclin-like arabinogalactan proteins FLA3 in *Arabidopsis* participated in pollen intine formation and may be involved in cellulose deposition [66], and that AGPs were involved in cell wall cellulose and pectin deposition in *Arabidopsis* embryos [18]. For in vitro culture of *Arabidopsis* ovules, 50 μM β-GlcY treatment affected cellulose and pectin deposition, leading to abnormal cell division and cotyledon formation during embryo development. All these results suggest that AGPs play important roles in cell wall organization and cell division in reproductive plant development, though the detailed mechanisms of AGPs functions are future topics for study.

**Figure 10:**
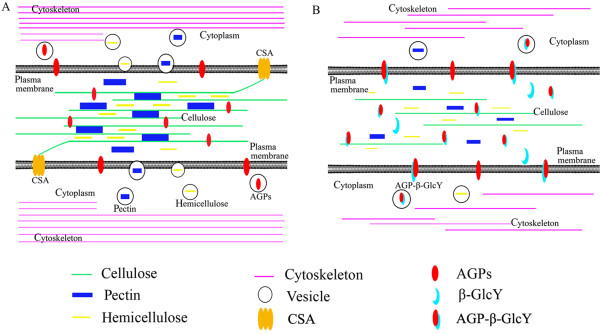
**A hypothetical model for how AGPs involve in early proembryo cells division. (A)** A possible *in vivo* model of cell plate formation. Cellulose synthesized by a complex of cellulose synthases (CSAs) on plasma membrane. Pectin and hemicellulose synthesized and secreted through the endomembrane system. Some types of AGPs anchor to membrane and some were directly secreted into cell wall space. Cytoskeleton networks transport endocytic vesicles to the correct site under the plasma membrane. **(B)** A possible in vitro model under β-GlcY treatment. β-GlcY reagent binds with AGPs to form an AGP-β-GlcY complex. Cell wall components and cytoskeleton network could be disorganized with β-GlcY treatment, which affects new cell plate formation.

Based on the previous [38,59,65] and present studies, a model of AGP involvement in cell plate formation was proposed (Figure 10). AGPs are abundant cell-surface proteins in plants and any AGP changes may affect other cell surface components or induce cytoskeletal abnormality [27,60,67]. In the zygotic embryogenesis, the loss of AGPs profoundly alters the first zygotic division and disarranges the proembryo pattern formation [15]. Our model suggests that after AGPs were bound by β-GlcY regent then might inhibit the interactions of AGPs with other molecules, thus may directly or indirectly affect cytoskeleton localization and disturb cytoskeleton-dependent transfer such as endocytotic delivery, and then further effects cell plate formation. Another possible model involved in this process is that AGP-Yariv complexes may disorder the assembly of the new cell wall and plasma membrane components, such as cellulose synthase (CSAs) [65]. Previous results in *Arabidopsis* have shown that the primary cell walls formation was effected and the cell elongation in the embryo axis was severely impaired in the null mutant of *cellulose synthase A*[68]. In this case, the AGPs may have two important responsibilities: one as signaling molecules and the other as structural components. However, the two possible functions of AGPs in zygotic division still need to be corroborated by more experimental evidence.

## Conclusions

Here, we used in vitro zygote culture and series of meticulous cell biology techniques to investigate the AGPs roles in tobacco proembryo cell division. In tobacco, for the first time, we carried out the detailed observation on each cell division event of zygote and proembryo, and compared the subtle differences between tobacco proembryo division patterns and those of *Arabidopsis*. In in vitro cultured proembryos, we found that in the case of AGPs were specific binding with β-GlcY and lost their functions, the cell divisions of zygotes and proembryos were disordered. Furthermore, we used fluorescent labeling approaches combined with ultrathin sections to observe the distribution of AGPs, cell wall materials such as pectin and cellulose, endocytic vesicles and other cell-organelles. The results showed that the cell plate specific location of AGPs, cellulose and endocytic vesicles were reduced or disorganized by the addition of an AGPs inhibitor, β-GlcY. In addition, the morphology and distribution of mitochondria and endoplasmic reticulum were both affected. Besides above findings, interestingly, we also found that low- and high-esterified pectins located in opposite positions of zygotes and proembryos and this polarity was hardly affected by β-GlcY. Based on our results, we may conclude that the cell plate positions decision and new cell wall materials location in vitro cultured zygotes and proembryos were both affected by β-GlcY treatment. These results imply that AGPs may contribute to the formation of the new cell plate, and suggest that AGPs play roles in some important developmental events of tobacco, including zygotic division and proembryo pattern establishment. Further molecular and genetic analysis will promote more promising research on mechanism of AGPs in plant development.

## Abbreviations

AGPs = Arabinogalactan proteins; β-GlcY = β-glucosyl Yariv; BSA = Bovine serum albumin; CLSM = Confocal laser scanning microscopy; CSAs = Cellulose synthase; CW = Calcofluor white; DAP = Days after pollination; DAPI = 4′,6-diamidino-2-phenylindole; DiOC_6_(3) = 3,3′-dihexyloxacarbocyanine iodide; DMSO = Dimethyl sulfoxide; FLA = Fasciclin-like arabinogalactan proteins; FM4-64 = N-(3-triethylammoniumpropyl)-4- (p-diethylam-inophenylhexatrienyl)-phridinium-2Br; HG = Homogalacturonan; PHP = Phragmoplast; PMEs = Pectin methylesterases; PPB = Preprophase band; RG-I = Rhamnogalacturonan-I; SG = Substituted galacturonans; ZGA = Zygote gene activation.

## Authors’ contributions

MY carried out the experiment, participated in the design of the study and drafted the manuscript. JZ conceived of the study, and participated in its design and helped to draft the manuscript. Both authors read and approved the final manuscript.
